# The Subjective Index for Physical and Social Outcome (SIPSO) in Stroke: investigation of its subscale structure

**DOI:** 10.1186/1471-2377-10-26

**Published:** 2010-04-26

**Authors:** Paula Kersten, Ann Ashburn, Steve George, Joseph Low

**Affiliations:** 1 School of Health Sciences, University of Southampton, Highfield, Southampton, UK; 2 School of Medicine, University of Southampton, Southampton, UK; 3 UCL Medical School, University College London, London, UK

## Abstract

**Background:**

Short and valid measures of the impact of a stroke on integration are required in health and social settings. The Subjective Index of Physical and Social Outcome (SIPSO) is one such measure. However, there are questions whether scores can be summed into a total score or whether subscale scores should be calculated. This paper aims to provide clarity on the internal construct validity of the subscales and the total scale.

**Methods:**

SIPSO data were collected as part of two parallel surveys of the met and unmet needs of 445 younger people (aged 18-65) with non-recent stroke (at least one year) and living at home. Factor, Mokken and Rasch analysis were used.

**Results:**

Factor analysis supported a two factor structure (explaining 68% of the variance) as did the Mokken analysis (overall Loevinger coefficient 0.77 for the Physical Integration subscale; 0.51 for the Social Integration subscale). Both subscales fitted the Rasch model (P > 0.01) after adjusting for some observed differential item functioning. The 10-items together did not fit the Rasch model.

**Conclusions:**

The SIPSO subscales are valid for use with stroke patients of working age but the total SIPSO is not. The conversion table can be used by clinicians and researchers to convert ordinal data to interval level prior to mathematical operations and other parametric procedures. Further work is required to explore the occurrence of bias by gender for some of the items.

## Background

Between 174 and 216 people per 100,000 per year suffer a stroke in the UK[1]. Of these about a third will have long-term disability[2]. The advent of better treatment, such as thrombolysis and improvements in acute rehabilitation services, means that more people will survive a stroke. A good quality of life after stroke, maximising independence, well-being and choices, is therefore an important focus for rehabilitation services [3,4]. Measuring such outcomes can be carried out by health care professionals, using tools such as the Barthel Index or the National Institutes of Health Stroke Scale. However, the impact of a stroke upon an individual's life is more appropriately measured by patients themselves. A candidate measure for this area of interest is the Stroke Impact Scale (SIS), which contains a domain measuring social participation [5], but also seven other domains. Thus, the SIS is rather long, placing significant burden on the participant in terms of completion. A much shorter scale, the Subjective Index of Physical and Social Outcome (SIPSO) (10 items) has a focus on physical and social integration after a stroke. The SIPSO was developed using extensive qualitative work [6] and validity and reliability has been shown to be good, when examined with traditional psychometric methods [7-9]. The SIPSO is much shorter than health status measures frequently used in stroke[10]. Thus, this shorter stroke specific measure is worthy of further exploration for use in clinical practice and research. The measure has been used in studies exploring unmet needs amongst people with stroke [11,12], the benefits of a community based exercise and education intervention [13] and a community ambulation intervention[14]. Although the scale has been shown to consist of two subscales, a physical and a social integration subscale [8], the originators have also proposed that a total score can be used[9]. They base this on a somewhat lower Cronbach alpha for the Social Integration subscale, even though this is acceptable for individual use (0.82) and somewhat lower correlations for items 6 and 10 with this subscale (though acceptable with correlation values of 0.67 and 0.74 respectively and greater than the correlation with the physical integration subscale). Items 6 and 10 measure how often someone feels bored and how s/he feels about appearing in public. Thus, there is a conflict between earlier statements that the scale consist of two subscales [8,9] and that the SIPSO also can be used in its totality [9] and at present total SIPSO scores are used in research [13,14]. Further, researchers use parametric analyses to analyse SIPSO data. Since the measure produces ordinal data it would be of use if an interval transformation could be produced. Interval transformations can be produced if the scale fits the Rasch model.

Rasch analysis is useful in testing whether items from a scale measure a unidimensional construct [15,16], which is required to justify the summation of scores. Rasch analysis transforms ordinal scores to the logit scale and thus to an interval-level measurement [15,16]. Furthermore, fitting data to the Rasch model allows for a detailed examination of the internal validity of the measure.

The aims of this paper are therefore twofold: (1) to provide clarity on the internal construct validity of the subscales and the total scale using factor analysis, Mokken analysis and Rasch analysis, and (2) to provide an interval conversion table if the SIPSO is found to meet the requirements of the Rasch model.

## Methods

The study design and recruitment procedures have been described in detail elsewhere[7]. Briefly, SIPSO data were collected as part of two parallel surveys of younger people (aged 18-65) with non-recent stroke (at least one year ago) and living at home. The studies aimed to measure met and unmet needs. Recruitment occurred via registers maintained by national stroke centres [11] and Young Stroke groups affiliated to the Stroke Association of England and Wales[12]. People were excluded if they had a diagnosis of subarachnoid haemorrhage, had other disabling illnesses (e.g. rheumatoid arthritis or multiple sclerosis) or lived in residential care. Clinicians in charge of the stroke registers [11] and Young Stroke group coordinators [12] checked for eligibility. Eligible people were sent the SIPSO as part of the Southampton Needs Assessment Questionnaire for People with Stroke (SNAQs) and asked to return the completed forms to the researcher in Southampton. Up to two follow-up attempts were made to contact non-responders (three weeks apart).

### Data Analysis

We used factor analysis, with parallel analysis to determine the significant eigenvalues [17], to examine the structure of the SIPSO and Mokken analysis to determine if the SIPSO was a valid ordinal scale [18-21]. Mokken scaling determines how likely it is that an item will be endorsed (item difficulty) and the amount of construct a person has (in this case level of integration). It assumes that a person with a certain amount of the construct (integration) will give a positive response to an item that is easier to endorse than his or her level of integration and a negative response to an item that is more difficult to endorse. It then tests this notion against the probability that the opposite will occur. Thus, Mokken scaling determines if a non-parametric probabilistic Guttman-style relationship exists in the data. Loevinger *H*-coefficients greater than 0.3 for individual items and the (sub) scale(s) as a whole were deemed acceptable of the probabilistic relationship.

Rasch analysis is a parametric probabilistic version of Guttman Scaling [16,22]. It is a simple logistic model, which assumes that more able people (in this case with more integration) are more likely to answer all items correctly (in this case give a more favourable response) and that easier items are more likely to be answered correctly (endorsed) by all. The interpretation of Rasch analysis has been explained in detail by others[23]. Briefly, fit to the Rasch model is acceptable when the summary chi-square interaction statistic is non-significant, showing no deviation from model expectation; where item and person summary fit statistics show a mean of zero and standard deviation of one; where individual items show non-significant chi-square fit statistics (Bonferroni adjusted), and where individual item and person residuals are within the range of +/- 2.5. Each item is examined to check that log-transformed item scores generated from the response choices reflect the increasing or decreasing latent trait to be measured. For example, a person scoring high on the subscale (good integration) should be more likely to tick the response option 4 (a positive response) than 0 (a negative response) on items which have been estimated as easy to endorse. Thresholds are the points where the probabilities of a response of either 0 or 1, and 1 or 2 (and so forth) are equally likely. If the SIPSO categories reflect increasing amount of integration, then we would expect thresholds defining the categories to be ordered along the trait accordingly. For disordered items categories can be collapsed.

In addition, the scale is expected to show invariance across key groups (e.g. gender). This requirement, also called absence of Differential Item Functioning (Dif), tests the requirement that people from different groups, with equal amounts of the underlying trait under investigation, respond to the item in the same manner; this is indicated by a non-significant ANOVA of the residuals where the key group is the main factor. Dif was examined for key groups including gender, age, centre, sample, and time since stroke.

Data should also be locally independent, in other words people's item responses should depend only on their trait level, not on their responses to other test items. This is examined with inter-item residual correlations, which should be below 0.30.

An independent t-test is used to examine if the scale is unidimensional. This tests whether any subset of items measures the same thing as another subset of items, using t-tests. If the 95% confidence interval of t-tests includes 5% unidimensionality is supported.

A reliability index, the Person Separation Index (PSI), is also calculated. The Person Separation Index (PSI) is similar to the Cronbach alpha but is derived from the linear estimates of the person's ability[22]. In a previous publication we demonstrated the reliability of the SIPSO with Cronbach's alphas of 0.93 for the Physical Integration subscale and 0.82 for the Social Integration subscale[7]. Targeting of the scale to the sample is also explored visually with person-item threshold maps.

Where data fit the model the manifest raw score from summated items can be transformed into interval scale measurement[24]. Bonferroni corrections were applied throughout the analysis to allow for multiple testing (P < 0.01)[25].

Mokken and Rasch analysis were conducted separately for the two subscales and for the SIPSO in its entirety to explore the internal construct validity of the subscales and the total SIPSO.

Factor analysis and all descriptive analyses were conducted using SPSS version 15[26]. Mokken scale analysis was undertaken with procedure 'msp' within STATA[27]. Rasch analysis was conducted using RUMM2020 software[28].

### Ethics

The study was approved by the South West Multi-Centre Research Ethics Committee.

## Results

In total 445 people took part (57% male, 39% female, 4% not declared). Their mean age was 53.7 (SD 9.0) and on average they had their stroke 3.5 years before we contacted them (SD 3.7, range 1-27).

### Factor analysis

The SIPSO items were subjected to a Principal Component Analysis. Suitability of data for this analysis was confirmed by correlations above 0.30, a Kaiser-Meyer-Oklin value of 0.91 (above the recommended value of 0.6) and a significant Bartlett's Test of Sphericity. Two eigenvalues were greater than those produced by a Monte Carlo Parallel Analysis, together explaining 68% of the variance. This supports the originally proposed two factor structure. With a correlation of 0.56 between the two factors an Oblimin Rotation was conducted, which also supported the two-factor structure. Table 1 and 2 display the Pattern Matrix and Structure Matrix.

**Table 1:** Structure Matrix SIPSO

Items	Component	
	**1**	**2**

Item 4	.899	.529

Item 2	.889	.502

Item 1	.866	.415

Item 5	.857	.485

Item 3	.848	.660

Item 8	.539	.854

Item 7	.351	.822

Item 6	.406	.748

Item 10	.536	.683

Item 9	.434	.666

**Table 2:** Pattern Matrix SIPSO

Items	Component*	
	**1**	**2**

Item 1	.919	

Item 2	.882	

Item 4	.876	

Item 5	.851	

Item 3	.697	

Item 7		.908

Item 8		.801

Item 6		.756

Item 9		.614

Item 10		.557

### Mokken analysis

The Physical and Social subscales were supported by the Mokken analysis with an overall Loevinger of 0.77 for the Physical Integration subscale and 0.51 for the social integration subscale. The total scale was also subjected to Mokken analysis and we found an overall Loevinger of 0.55, which is above the accepted cut off value of 0.30. However, Mokken analysis assumes the scale is unidimensional and the data were therefore further subjected to Rasch analysis.

### Rasch analysis

**Figure 1:**
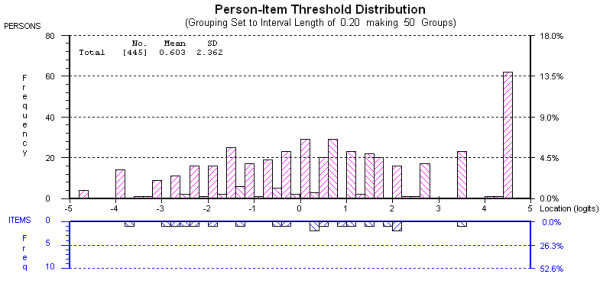
**Person Item Threshold Map Physical Integration subscale**.

The Physical Integration subscale initially did not fit the Rasch model as indicated by the significant chi-square value and uniform Dif by gender for items 1 (difficulty dressing) and 5 (independence in local neighbourhood) (Table 3, analysis 1). Combining items 1 and 5 into a subtest dealt with the Dif satisfactorily and the subscale was found to fit with a non-significant chi-square, sufficient fit statistics, local independence, unidimensionality, ordered item thresholds, and item fit (analysis 2). Targeting of the subscale to the sample was good (Figure 1). We were able to produce a conversion table for the subscale (Table 4). Reliability was high with a PSI of 0.93, (similar to the Cronbach alpha of 0.93), suggesting the subscale can distinguish between 4-5 groups of people[29].

**Table 3:** SIPSO Rasch analysis results

Analysis number	Item fit residual		Person fit residual		χ^2 ^interaction		PSI	Unidimensionality Independent t-test (95% CI)
	**Mean**	**SD**	**Mean**	**SD**	**Value (df)**	**P**		

Physical Integration								

1	0.145	1.271	-0.319	0.891	50.96 (30)	0.009	0.93	2.9% (0.7 to 5.2)

2	0.033	1.544	-0.351	0.881	37.87 (24)	0.036	0.93	1.6% (-0.6 to 3.8)

Social Integration								

3	0.340	1.787	-0.329	1.059	49.70 (30)	0.013	0.82	1.7% (-0.4 to 3.7)

4	0.188	2.824	-0.365	1.016	38.02 (24)	0.035	0.83	2.1% (0.1 to 4.2)

5	-0.097	2.413	-0.527	0.891	8.98 (12)	0.705	0.85	2.2% (0.1 to 4.3)

Total SIPSO								

6	-0.109	3.097	-0.310	1.198	179.62 (60)	<0.001	0.91	16.8% (14.8 to 18.9)

7	-0.261	2.801	-0.305	1.198	171.25 (60)	<0.001	0.91	14.5% (12.4 to 16.6)

8	-0.595	3.181	-0.382	1.064	158.19 (48)	<0.001	0.90	9.2% (7.1 to 11.3)

9	-0.328	2.619	-0.341	0.946	118.85 (36)	<0.001	0.92	4.7% (2.5 to 6.9)

10	0.115	1.323	-0.313	0.871	58.49 (30)	0.001	0.92	3.5% (1.3 to 5.7)

11	-0.041	1.529	-0.353	0.863	41.12 (24)	0.016	0.92	2.7% (0.5 to 4.9)

**Table 4:** Conversion table for the Physical and Social Integration subscales

Subscale raw score (ordinal)	Physical Integration Rasch log-transformed score (interval)	Social Integration Rasch log-transformed score (interval)
0	0.00	0.00

1	2.05	1.79

2	3.52	3.02

3	4.55	3.89

4	5.40	4.57

5	6.18	5.17

6	6.94	5.75

7	7.65	6.30

8	8.36	6.85

9	9.05	7.46

10	9.70	8.06

11	10.37	8.72

12	11.04	9.45

13	11.73	10.24

14	12.47	11.10

15	13.22	12.05

16	14.05	13.13

17	15.01	14.33

18	16.17	15.78

19	17.77	17.61

20	20.00	20.00

**Figure 2:**
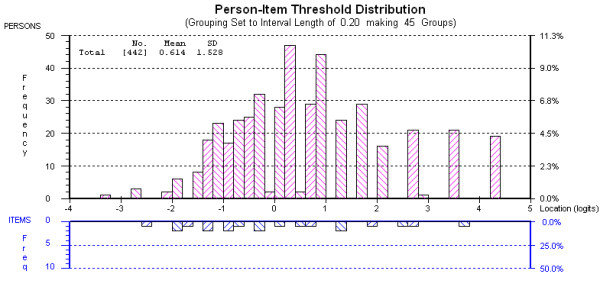
**Person Item Threshold Map Social Integration subscale**.

The Social Integration subscale also suffered from significant uniform Dif by gender (items 7 and 10; communication and appearance in public respectively) as well as sample (item 9; visiting friends) and subsequently did not fit the Rasch model (Table 3, analysis 3). Initially we created a subtest, combining items 7 and 10 and testing these against the remaining three items but the data again did not fit as other Dif was not dealt with (analysis 4). Therefore, another subset was created combining item 9 with items 6 and 8, which visually appeared to be biased in the opposite direction (though not significantly so). This resulted in satisfactory fit to the model and unidimensionality (analysis 5). With two (super) items remaining, reliability was sufficient for individual use (PSI 0.85) and the subscale can distinguish between 3-4 groups of people [29]. Again this value was similar to the Cronbach alpha of 0.82. As above, a conversion table was produced (Table 4) and targeting was good (Figure 2).

To test the assertion that the SIPSO makes a valid scale in its entirety all 10 SIPSO items were fitted to the Rasch model. The data deviated significantly from the Rasch model. Table 3 (analysis 6) shows a very high item fit residual standard deviation and chi-square value. Four items were disordered. Collapsing categories did not result in satisfactory fit (analysis 7); two items had high negative fit residuals (items 3 & 4), two had high positive fit residuals (items 6 & 7) and five had significant p-values (items 2, 3, 4, 6, 7). High positive fit residuals are indicators that they do not belong to the construct under investigation and should be deleted. After deletion of items 6 and 7, a further two showed significant misfit (high positive residuals, items 8 & 9, analysis 8). We deleted these two items (one at a time), at which point item 10 showed significant misfit (analysis 9) and had to be deleted. The resulting five-item subscale contained all the items belonging to the physical integration subscale and as before this showed Dif by gender (analysis 10). Creating the subtest as described above resulted in satisfactory fit to the Rasch model (analysis 11). The slightly different fit statistics between analysis 2 and 11 arises from the fact that in the latter data was rescored.

## Discussion

The complementary use of factor analysis, Mokken scaling and Rasch analysis allowed us to conduct a thorough investigation of scaling properties of the SIPSO. The three analyses provided incremental evidence for the validity of the two SIPSO subscales: Factor analysis confirmed the two-factor subscale structures proposed by its originators [8]; Mokken analysis showed that the two subscales were valid ordinal scales; and Rasch analysis demonstrated that they conformed to the most stringent requirements of measurement and were unidimensional. Therefore, we are confident that the two subscales can be used.

The Mokken scaling showed an acceptable *H*-Loevinger Coefficient for the total SIPSO. Mokken scaling determines if an ordinal scale has been constructed but assumes that the scale is unidimensional. Unidimensionality is a requirement for summating any set of items and this is part of the basic science of measurement. Factor analysis and Rasch analysis showed that the 10 SIPSO items did not form a valid, unidimensional scale. The findings from the former found two significant eigenvalues and the latter demonstrated misfit to the Rasch model when all items were tested against the Rasch model. Rasch analysis is strict in terms of satisfying the requirement for transformation to interval scaling [30,31]. The iterative process of Rasch analysis requires unidimensionality tests to be done at each stage. Thus, factor analysis and Rasch analysis provide their own hierarchical ordering of scalability with the assumption of unidimensionality and finally the potential for interval scale transformation. Thus, the three analyses provided incremental evidence of the two subscales, but not the total SIPSO.

The Rasch model has specific properties associated with fundamental measurement, specifically, the raw score as a sufficient statistic, and the separation of person and item parameters[15]. The former is important as clinicians and others add up the set of responses to make a total score and use these to calculate change scores. As ordinal scales do not support such mathematical operations this is inappropriate. The Rasch model is the only Item Response Theory model that provides an interval scale transformation of the data. As our subscale data fit the Rasch model we were able to produce a conversion table. This table will aid clinicians and researchers in the conversion of the raw data into interval level data for the purpose of mathematical procedures such as summing subscale totals, calculating change scores and for parametric statistical analyses.

The SIPSO is relatively new and there are not many publications reporting its use even though it uniquely measures stroke specific physical and social outcome. There are no other measures that enable the evaluation of physical and social outcome in stroke although there are self-reported health related quality of life (HRQOL) scales, which include aspects of these domains. For example, frequently cited [10] stroke specific HRQOL measures include the Stroke-Specific Quality of Life Scale [32] and the Stroke Impact Scale[5]. However, these measures are much longer, contain other domains and do not have this specific focus on physical and social integration. A direct comparison between these longer HRQOL measures and the SIPSO would enable the comparison of their research and clinical utility. In addition, to compare findings in stroke populations with other groups of patients it will be useful to also include a generic measure in research.

The sample size (n = 445) for the study was estimated for the two parallel needs studies [11,12]. A retrospective sample size calculation for the Rasch analysis took into account that in order to be able to report the transformation of ordinal to interval scores normally requires a minimum of 250 cases, or 20 times the number of items, whichever is the greater. Therefore, with a ten item scale this requires 200 cases. As our sample included 445 people this was more than sufficient.

This study included only patients aged 18 to 65. Only 25% of people experience a stroke are younger than 65[2]. Therefore, our study makes no claims about the appropriateness of the SIPSO in older patients although this has been established by others [6,8,9]. The SIPSO does not include items that are only specific to people of a certain age. In addition, a key characteristic of Rasch analysis is that of *specific objectivity*. This is the estimation of item difficulty (or endorsability) independent of the distribution of the person estimates (in this case the amount of physical or social integration someone experiences) in the particular group of patients, and vice versa[15]. In other words, Rasch analysis is said to enable sample-free estimates of item difficulty. We can therefore conclude that the SIPSO subscales are valid and unidimensional for stroke patients, irrespective of their age.

The SIPSO was completed by post and the overall response rate in the two surveys was 53% [11,12] despite strategies that have been shown to improve response rates such as follow-up letters together with the questionnaire and the supply of self-reply envelopes [33,34]. However, this response rate tends to be in line with other postal surveys[35]. In a survey of people discharged from UK hospitals after their stroke it was shown that of those who rated their care as very poor to fair immediately following discharge, fewer responded to the follow-up survey one year later than those who had said the care was good to excellent[36]. Whether or not our surveys incurred this self-selection bias is impossible to say as we did not the opportunity to collect such data. Non-responders in our first survey were similar to responders in terms of their age and gender[11]. As for the second survey we were unable to record data on non-responders we are not able to comment on differences between responders and non-responders[12]. For future studies it would be useful to collect more data on non-responders, though the Research Governance Framework for Health and Social Care [37] and the Data Protection Act pose significant challenges in achieving such ambitions.

## Conclusions

The SIPSO subscales are valid for use with stroke patients of working age but the total SIPSO is not. The conversion table can be used by clinicians and researchers to convert the raw data to interval level data after which mathematical operations (e.g. summing up subscale scores, calculating change scores) and other parametric procedures can be performed. Further work is required to explore the occurrence of bias by gender for some of the items.

## Competing interests

The authors declare that they have no competing interests.

## Authors' contributions

PK, AA and SG conceived of the study, were responsible for its design and coordination and helped to draft the manuscript. JL collected the data. PK performed the analyses and drafted the manuscript. All authors read and approved the final manuscript.

## Pre-publication history

The pre-publication history for this paper can be accessed here:

http://www.biomedcentral.com/1471-2377/10/26/prepub
